# Prevalence of Hepatocellular Carcinoma in HIV Patients Co-infected or Triple Infected With Hepatitis B and Hepatitis C in a Community Hospital in South Bronx

**DOI:** 10.7759/cureus.26089

**Published:** 2022-06-19

**Authors:** Shehriyar Mehershanhi, Asim Haider, Sameer Kandhi, Haozhe Sun, Harish Patel

**Affiliations:** 1 Gastroenterology and Hepatology, BronxCare Health System, Bronx, USA; 2 Internal Medicine, BronxCare Health System, Bronx, USA

**Keywords:** south bronx, co-infection, hepatocellular carcinoma (hcc), human immunodeficiency virus (hiv), hepatitis b, hepatitis c

## Abstract

Background

Human immunodeficiency virus (HIV), hepatitis B virus (HBV), and hepatitis C virus (HCV) share common modes of transmission; hence HBV and HCV infection are more prevalent among HIV patients. The co-infection with HIV/HBV, HIV/HCV, or HIV/HBV/HCV carries significant morbidity, with higher progression rates to end-stage liver disease or hepatocellular carcinoma (HCC).

Methods

We conducted a retrospective study among HIV adult patients co-infected with HBV or HCV and those with HCV, HIV, and HBV triple infection enrolled in the outpatient clinic of BronxCare Hospital between the years 2010 and 2021. Records were reviewed to obtain demographic data, including age and sex, hepatitis B surface antigen (HBsAg), anti-HCV antibodies, and CD4 T-lymphocyte count test results. Male and female patients ≥18 years with confirmed HIV by double enzyme-linked immunoassay (ELISA) and western blot, who underwent serology testing for both HBsAg and anti-HCV, were included in the study.

Results

In this study, 11355 HIV patients were included, comprising 7020 (61.8%) males and 4335 (38.2%) females. A total of 410 (3.6%) were hepatitis B positive, 1432 (12.6%) were hepatitis C positive, and 127 (1.1%) were both hepatitis B and C positive. Fifty-two (0.5%) patients were diagnosed with HCC. The majority of the patient with HCC (50%, n =26) were hepatitis C serology positive (p<0.001) while 9.6% (n=5) were positive for both hepatitis C and hepatitis B (p<0.001).

Conclusion

HIV/HBV/HCV triple-infected patients had a lower rate of HCC compared to HIV/HCV co-infected patients. HIV without hepatitis C or hepatitis B is an independent risk factor for HCC.

## Introduction

Human immunodeficiency virus (HIV) infection remains one of the most severe worldwide health threats of our times. As per global HIV and acquired immunodeficiency syndrome (AIDS) statistics, in the year 2020, a total of 37.7 million (30.2 million-45.1 million) people were known to be living with HIV. Also, an additional 6.1 million people are estimated to be living with HIV but are undiagnosed or unaware of their illness [1]. Hepatitis B virus (HBV) and hepatitis C virus (HCV) infect liver cells and lead to chronic liver diseases such as hepatitis, cirrhosis, and hepatocellular carcinoma (HCC). The World Health Organization (WHO) Global Viral Hepatitis report estimates around 296 million people living with chronic hepatitis B infection in 2019, with 1.5 million new infections each year and 820,000 deaths from the infection annually [2]. Similarly, HCV is a significant cause of hepatitis with a propensity for chronicity. In 2019, around 58 million people were estimated to have chronic hepatitis C virus infection, with about 1.5 million new infections occurring per year and 290,000 deaths due to the infection or from its complications annually [3].

Available literature highlights the rapid progression of HBV and HCV in individuals co-infected with HIV [4-5]. These patients usually have worsened prognosis and shortened life span, with a significant proportion of patients with HIV/HBV or HIV/HCV co-infection ending up in end-stage liver disease or HCC compared to individuals with chronic hepatitis B or hepatitis C mono-infection [4-5]. Also, both these viruses have been documented to be more prevalent in HIV-positive patients with an associated increase in liver-related morbidity and mortality, which is twice as high in HIV/HBV co-infected individuals than those with HIV/HCV co-infection [6]. About 3-6 million people living with HIV are co-infected with HBV and 4-5 million people with HCV [6]. Such high rates of co-infection can be attributed to the identical routes of transmission of these viruses, which includes parenteral (blood and blood products and unsafe injection practices amongst people who inject drugs or tattoos), and sexual routes (men who have sex with men, heterosexual persons with multiple sex partners) [7]. Also, the prevalence of these co-infections varies with geographical area, risk groups, and type of exposure involved, with the highest rates of prevalence being reported in South Africa [8], Nigeria [9], and India [10]. However, despite the available extensive literature on HIV/HBV or HIV/HCV co-infections, little is known about outcomes, prognosis, and treatment of HIV, HBV, and HCV triple infections. Also, studies evaluating the incidence/progression of HCC in patients with triple infected patients with HIV/HBV/HCV are sparse.

Our study aims at investigating the prevalence of HIV patients with HBV/HCV triple infection and those with HIV/HCV or HIV/HBV co-infection along with the prevalence of HCC in patients with co-infections or triple infections with these viruses and demonstrates the relationship between the incidence of HCC with different CD4 counts stratified by hepatitis B or hepatitis C positivity or both. The study was carried out at the outpatient clinic of a tertiary hospital serving the population of the South Bronx. Bronx County has one of the highest rates of HIV per 100,000 population, three times above the state average and six times the national average [11]. Also, annual rates for chronic hepatitis B and chronic hepatitis C infection are several folds higher than the national average of the United States [12]. This study will be critical in guiding decisions; early initiation of antiviral treatment therapy can be best prescribed in co-infected patients, leading to the appropriate management of HIV and viral hepatitis. It can also aid in the early detection of HCC and thereby guide the necessary treatment approaches.

## Materials and methods

We conducted a retrospective study among adults with HIV patients with HBV and HCV infection and those with HIV and HCV, HIV, and HBV co-infection enrolled in the outpatient clinic of BronxCare Hospital between the years 2010 and 2021. Data within the electronic medical records (EMR) was evaluated, including hospital and outpatient International Classification of Diseases, ninth and tenth (ICD-9 & ICD-10) demographic, diagnoses, and laboratory results. The BronxCare Health System Institutional Review Board (IRB) reviewed and approved the study with reference number # 9121908.

The medical and laboratory records of 11,355 adult HIV-positive patients were reviewed to obtain demographic data, including age and sex, hepatitis B surface antigen (HBsAg), antibodies to HCV (anti-HCV), and CD4 T-lymphocyte count test results. Male and female patients were ≥18 years with confirmed HIV seropositivity by double ELISA and Western blot, who underwent serology testing for both HBsAg and anti-HCV, irrespective of their liver condition. Pediatric patients were excluded.

HIV, HBV, and HCV-infected patients had: (1) HIV (chronic HIV ICD-9/10 diagnoses, positive HIV antibody result, or CD4 count); (2) chronic hepatitis C (chronic HCV ICD-9/10 diagnoses, detectable HCV antibody, or positive qualitative HCV RNA result), and (3) chronic hepatitis B (chronic HBV ICD-9/10 diagnoses, 2 positive HBV surface antigen results)

HIV and HCV-infected patients had: (1) HIV (chronic HIV ICD-9/10 diagnoses, positive HIV antibody result, or CD4 count); (2) chronic hepatitis C (chronic HCV ICD-9/10 diagnoses, detectable HCV antibody or positive qualitative HCV RNA result), and (3) no chronic HBV ICD-9/10 diagnosis, 2 positive HBV surface).

HIV and HBV-infected patients had: (1) HIV (chronic HIV ICD-9/10 diagnoses, positive HIV antibody result, or CD4 count); (2) chronic hepatitis B (chronic HBV ICD-9/10 diagnoses, two positive HBV surface antigen results), and (3) no chronic HCV ICD-9/10 diagnoses, detectable HCV antibody, or positive qualitative HCV RNA result.

HCC patients had: (1) HCC ICD-9/10 diagnoses, (2) typical radiologic aspect in at least one image exam (CT or MRI) following the criteria established by the European Association for the Study of the Liver (EASL), and (3) pathology diagnosis for HCC.

The statistical analysis was performed using IBM SPSS (Statistical Packages for the Social Sciences) version 19 (IBM Corp, Armonk, NY). We reported the continuous variable with the mean and standard deviation. Frequencies and percentages were reported for categorical variables. Continuous variables were compared using one-way analysis of variance (ANOVA) and dichotomous variables were compared by chi-square analysis using the Pearson test.

## Results

There were 11355 HIV patients in the study, of which 61.8% (n=7020) were male patients and the remaining 38.2% (n=4335) were females. The majority of them were men. The mean age of the males was 54.12 (+13.55) versus those of females was 54.1 (+12.61). There was no statistical difference between male and female mean age (p = 0.92). The median range of CD4 count for males was 393 and for females, it was 475. A total of 410 (3.6%) were hepatitis B positive and 1432 (12.6%) were hepatitis C positive. There is a significant correlation between gender and the hepatitis B positive patient (p=0.045) and those with hepatitis C positive serology (p<0.001). There was no statistically significant difference in the gender distribution of the patient with double infection of hepatitis B and hepatitis C (p=0.321). Fifty-two (0.5%) of patients were diagnosed with HCC (Table 1).

**Table 1:** Gender distribution of various demographic parameters in HIV-positive patients HIV: human immunodeficiency virus

	Gender		
	Female	Male	P-Value
	(n=4335)	(n=7020)	
Age	54.1 (+13.55)	54.1 (+12.61)	0.92
CD4 count	475	393	0.009
Hepatitis B	137 (3.2%)	273 (3.9%)	0.045
Hepatitis C	436 (10.1%)	996 (14.2%)	<0.001
Hepatitis B and hepatitis C positive	42 (1%)	82 (1.2%)	0.321
Hepatocellular carcinoma	8 (0.2%)	44 (0.6%)	<0.001

Patients with HCC tend to be older, with a mean age of 66.6 years compared to those without HCC; this difference is statistically significant (p=<0.001). There was no difference in CD4 count between the group with HCC surveillance and the one with no HCC (Table 2).

**Table 2:** Correlation of hepatocellular carcinoma with hepatitis serology

	Hepatocellular Carcinoma		
	Absent	Present	p value
	(n=11303)	(n=52)	
Age	54.1(+13.2)	66.6(+7.7)	<0.001
Absolute CD4 count	482(+356)	476(+360)	0.943
hepatitis B positive and hepatitis C negative	285 (2.5%)	1(1.9%)	0.784
hepatitis C positive and hepatitis B negative	1282(1.3%)	26(50%)	<0.001
hepatitis B positive and hepatitis C positive	119(1.1%)	5(9.6%)	<0.001
Hepatitis B negative and hepatitis C negative	9617 (85.1%)	20(38.5%)	<0.001

We analyzes the data for patients with only hepatitis B positive (hepatitis B positive and hepatitis C negative) and only hepatitis C (hepatitis C positive and hepatitis B negative), and the number of patients was 286 (2.5%) and 1308 (11.5%). However, the presence of hepatitis B was not significantly associated with HCC. One-hundred twenty-four (124) patients (1.1%) had hepatitis B and hepatitis C mixed infection. The majority of the patients with HCC (50%, n =26) were hepatitis C serology positive and the presence of hepatitis C significantly correlates with the HCC. The presence of hepatitis C and hepatitis B contributed to around 9.6% (n=5), which was statistically significant. There were 20 (38.5%) patients with HCC and HIV with no prior hepatitis C or hepatitis B infection.

We grouped the study population into three groups based on the CD4 count of fewer than 200 cells/dL, 201 to 500 cells/dL, and those above 500 cells/dL. Hepatitis serology and the presence of HCC were compared (Table 3). There was no direct correlation between the CD4 count and HCC.

**Table 3:** Hepatocellular carcinoma in different CD4 groups along with hepatitis serology

	Hepatocellular Carcinoma					
	Absent			Present		
CD4 group	Hepatitis B +, Hepatitis C -	Hepatitis C +, Hepatitis B -	Hepatitis B +, Hepatitis C +	Hepatitis B +, Hepatitis C -	Hepatitis C +, Hepatitis B -	Hepatitis B +, Hepatitis C +
Under 200	66 (33.7%)	197 (29%)	30 (35.7%)		4 (33.3%)	2 (100%)
201 to 500	73 (37.2%)	242 (35.6%)	32 (38.1%)	1 (1.9%)	1 (8.3%)	
Above 501	57(29.1%)	240(35.3%)	22(26.2%)		7(58.3%)	

For patients with CD4 count > 500 cell/dL, HCC was present in those who were hepatitis C positive. This was statistically not significant with a p-value of 0.425. In patients with mixed hepatitis B and hepatitis C infection, HCC was more prevalent if the CD4 count was below 200 cell/dL. This was statistically not significant.

We wanted to analyze any difference in the HCC rate in patients with different CD4 counts, specifically for hepatitis C infection (Table 4). We noticed that in patients with hepatitis C, HCC was more prevalent in the extremes of the CD4 count, that is, 2% in those with CD4 below 200 cells/dL and 2.8% in those with a CD4 count above 500 cell/dL. However, we did not see any statistical difference to demonstrate the difference in the CD4 counts accounted for the presence or absence of HCC (Table 4).

**Table 4:** Demonstrates hepatocellular carcinoma in patients with different CD4 counts layered by hepatitis C positivity

		Under 200	201 to 500	Above 501	P-Value
Hepatitis C negative	Hepatocellular Carcinoma +ve	4 (0.4%)	1 (1%)	4 (0.2%)	0.49
	Hepatocellular Carcinoma -ve	1058 (99.6%)	1489 (99.9%)	1865 (99.8%)	
Hepatitis C positive	Hepatocellular Carcinoma +ve	4 (2%)	1 (0.4%)	7 (2.8%)	0.425
	Hepatocellular Carcinoma -ve	197 (98%)	242 (99.6%)	240 (97.2%)	

## Discussion

This study investigated possible differences between HCC prevalence in HIV-infected patients co-infected or triple infected with hepatitis B or hepatitis C compared with only HIV-positive patients. Age, hepatitis C co-infection, hepatitis B and C triple infection, and HIV patients with no hepatitis B or C were found to have HCC, which were clinically significant (p<0.001). Adult HIV-positive patients co-infected with HCV showed a higher risk of HCC than patients who had triple infection with hepatitis B and hepatitis C or had negative hepatitis B or C, 50%, 9.6%, and 38.5%, respectively. Giordano et al. reported that HIV patients with hepatitis C virus co-infection promotes the development of HCC five-fold. One of the largest longitudinal studies showed that the incidence rates of HCC in the HIV-only and co-infected groups were 0.20 and 1.32 per 1000 person-years, respectively [13]. Evidence suggests that HIV infection increases the risk of HCC and has a more aggressive clinical course when co-infected with HCV infection [14-15].

The first case of HCC in an HIV-positive patient without evidence of HCV or HBV infection was reported by Tanaka et al. [16]. Our knowledge of HCC outcomes, prognosis, and treatment in HIV-infected patients is scarcely known. We observed 38.5% of HIV patients with HCC with no prior hepatitis C or hepatitis B infection. HIV infection has been shown to be independently associated with a reduced survival rate in HCC patients compared to HCC in HIV-negative patients [17]. In HIV patients, qualitative and quantitative changes in the host's immune status are key factors for HIV-associated HCC [18]. It has been hypothesized that in HIV patients, HCC development, progression, and spread are secondary to a weakened anti-tumor immune response and tumor-associated inflammation [19].

Clifford et al. established a significant association for the development of HCC in patients with HIV/hepatitis B co-infected who had low CD4+ cells and were significantly associated with HCC [20]. We demonstrated that there was no direct correlation between the CD4 count and the HCC. We did notice that in patients with the HIV/hepatitis C co-infection, HCC was more prevalent in the extremes of the CD4 count, but it was statistically not significant. The largest single study of cancer risk in people with HIV/AIDS found no relationship between CD4+ cell count and HCC [21]. It has been hypothesized that HIV/HBV co-infected patient with low CD4 count is associated with a higher level of HBV replication and hastening progression to cirrhosis and HCC [22].

Conversely, there was no substantial evidence of an association between HIV/hepatitis B co-infection and HCC in our investigation. In 2018, a retrospective cohort study showed that HIV co-infection in chronic liver disease due to HBV showed no relation to the increase in HCC incidence [23]. In Western countries, there has been no significant increase in the incidence of HCC in patients co-infected with HIV and HBV. In part, as highly active antiretroviral therapy (HAART) and HBV treatment is readily available, this has shown to decrease mortality and morbidity. Another explanation could be that the patient has succumbed to other HIV-related opportunistic infections in a period too short to allow the development of HBV-induced HCC [24-25].

While the proportion of individuals with a combination of HIV, HBV, and HCV is very low in a general population, a systematic review and meta-analysis by Fahimeh et al. and Agarwal et al. showed a slight prevalence of triple infections (1.25%) and (1.83%) in people who injected drugs [26] and in blood donors [27], respectively. Our study showed 124 patients (1.1%) with triple infection in South Bronx, NY. On the contrary, HIV-HBV-HCV triple infection was more prevalent in injection drug user (IDU) patients from China and Myanmar (19.1% vs. 10.4%, P<0.005) [28]. Multiple studies with HIV, HBV, and HCV triple infection showed higher rates of hepatic decompensation, death rates, and pre-cancerous coexistence in those who did not receive anti-HBV treatment in combination with the HIV regime [29-30]. Hence, in our study, the risk of HCC in triple-infected patients was 9.6% compared to 50% in patients co-infected with HCV, which was statistically significant <0.001.

## Conclusions

HIV/HBV/HCV-infected patients had a lower rate of HCC compared to HIV/HCV co-infected patients. HIV without hepatitis C or hepatitis B is an independent risk factor for HCC, accounting for 38.5%, which was statistically significant at <0.001. There was no significant correlation between CD4 counts and HCC. This study helps bring awareness that even though triple infection has a low prevalence in communities, there is a risk of HCC in treated or untreated patients.

## References

[1] (2020). Global HIV & AIDS statistics - fact sheet. https://www.unaids.org/en/resources/fact-sheet.

[2] (2022). World Health Organization. Hepatitis B. https://www.who.int/news-room/fact-sheets/detail/hepatitis-b.

[3] (2019). World Health Organization. Global health observatory data. https://www.who.int/news-room/fact-sheets/detail/hepatitis-c.

[4] Greub G, Ledergerber B, Battegay M (2001). Clinical progression, survival, and immune recovery during antiretroviral therapy in patients with HIV-1 and hepatitis C virus coinfection: the Swiss HIV Cohort Study. Lancet.

[5] Cheng Z, Lin P, Cheng N (2021). HBV/HIV coinfection: impact on the development and clinical treatment of liver diseases. Front Med (Lausanne).

[6] Kourtis AP, Bulterys M, Hu DJ, Jamieson DJ (2012). HIV-HBV coinfection--a global challenge. N Engl J Med.

[7] Nnakenyi ID, Uchechukwu C, Nto-Ezimah U (2020). Prevalence of hepatitis B and C virus co-infection in HIV positive patients attending a health institution in southeast Nigeria. Afr Health Sci.

[8] Parboosing R, Paruk I, Lalloo UG (2008). Hepatitis C virus seropositivity in a South African Cohort of HIV co-infected, ARV naïve patients is associated with renal insufficiency and increased mortality. J Med Virol.

[9] Hamza M, Samaila AA, Yakasai AM, Babasha M, Borodo MM, Habib AG (2013). Prevalence of hepatitis B and C viruses’ infection among HIV-infected patients in a tertiary hospital in north-western Nigeria. Niger J Basic Clin Sci.

[10] Saravanan S, Velu V, Kumarasamy N (2007). Coinfection of hepatitis B and hepatitis C virus in HIV-infected patients in south India. World J Gastroenterol.

[11] (2019). HIV & AIDS Bronx County data. https://aidsvu.org/local-data/united-states/northeast/new-york/bronx-county/.

[12] (2017). Hepatitis A, B and C in New York City: 2017 annual report. Internet.

[13] Giordano TP, Kramer JR, Souchek J, Richardson P, El-Serag HB (2004). Cirrhosis and hepatocellular carcinoma in HIV-infected veterans with and without the hepatitis C virus: a cohort study, 1992-2001. Arch Intern Med.

[14] Puoti M, Bruno R, Soriano V (2004). Hepatocellular carcinoma in HIV-infected patients: epidemiological features, clinical presentation and outcome. AIDS.

[15] Bräu N, Fox RK, Xiao P (2007). Presentation and outcome of hepatocellular carcinoma in HIV-infected patients: a U.S.-Canadian multicenter study. J Hepatol.

[16] Tanaka T, Imamura A, Masuda G (1996). A case of hepatocellular carcinoma in HIV-infected patient. Hepatogastroenterology.

[17] Berretta M, Garlassi E, Cacopardo B (2011). Hepatocellular carcinoma in HIV-infected patients: check early, treat hard. Oncologist.

[18] Pinato DJ, Merli M, Dalla Pria A (2017). Systemic inflammatory response is a prognostic marker in HIV-infected patients with hepatocellular carcinoma. Oncology.

[19] Sulkowski M (2009). Hepatocellular carcinoma in HIV-infected patients comes of age: the convergence of epidemiology and treatment effectiveness. J Hepatol.

[20] Clifford GM, Rickenbach M, Polesel J (2008). Influence of HIV-related immunodeficiency on the risk of hepatocellular carcinoma. AIDS.

[21] Frisch M, Biggar RJ, Engels EA, Goedert JJ (2001). Association of cancer with AIDS-related immunosuppression in adults. JAMA.

[22] Colin JF, Cazals-Hatem D, Loriot MA (1999). Influence of human immunodeficiency virus infection on chronic hepatitis B in homosexual men. Hepatology.

[23] Marcon PD, Tovo CV, Kliemann DA, Fisch P, de Mattos AA (2018). Incidence of hepatocellular carcinoma in patients with chronic liver disease due to hepatitis B or C and coinfected with the human immunodeficiency virus: a retrospective cohort study. World J Gastroenterol.

[24] Smukler AJ, Ratner L (2002). Hepatitis viruses and hepatocellular carcinoma in HIV-infected patients. Curr Opin Oncol.

[25] Herrero Martínez E (2001). Hepatitis B and hepatitis C co-infection in patients with HIV. Rev Med Virol.

[26] Bagheri Amiri F, Mostafavi E, Mirzazadeh A (2016). HIV, HBV and HCV coinfection prevalence in Iran-a systematic review and meta-analysis. PLoS One.

[27] Agarwal N, Dubey U, Agarwal A, Jaiswal R (2011). Hepatitis B or hepatitis C: the bigger threat in multiple infected HIV positive blood donors. J Clin Diagnostic Res.

[28] Zhou YH, Liu FL, Yao ZH, Duo L, Li H, Sun Y, Zheng YT (2011). Comparison of HIV-, HBV-, HCV- and co-infection prevalence between Chinese and Burmese intravenous drug users of the China-Myanmar border region. PLoS One.

[29] Lo Re V 3rd, Wang L, Devine S, Baser O, Olufade T (2014). Hepatic decompensation in patients with HIV/Hepatitis B Virus (HBV)/Hepatitis C Virus (HCV) triple infection versus HIV/HCV coinfection and the effect of anti-HBV nucleos(t)ide therapy. Clin Infect Dis.

[30] Martín-Carbonero L, Teixeira T, Poveda E, Plaza Z, Vispo E, González-Lahoz J, Soriano V (2011). Clinical and virological outcomes in HIV-infected patients with chronic hepatitis B on long-term nucleos(t)ide analogues. AIDS.

